# Parameters sensitivity assessment and heat source localization using infrared imaging techniques

**DOI:** 10.1186/s12938-017-0403-2

**Published:** 2017-09-21

**Authors:** Maryam Rastgar-Jazi, Farah Mohammadi

**Affiliations:** Department of Electrical and Computer Engineering, Ryerson University, 350 Victoria St, Toronto, ON M5B 2K3 Canada

**Keywords:** Infrared imaging, Malignant cells, Artificial neural networks, Penne’s bioheat equation, Sensitivity

## Abstract

**Background:**

At present, infrared (IR) imaging is used both as a non-invasive and a non-ionizing technology. Using an IR camera, it is possible to measure body surface temperature in order to detect tumors and malignant cells. Tumors have a high amount of vasculature and an enhanced metabolism rate, which may result in an increase in body surface temperature by several degrees above its normal level.

**Methods:**

Using thermograms, it is possible to assess various tumor parameters, such as depth, intensity, and radius. Also, by solving for Penne's bioheat equation, it is possible to develop the analytical method to solve for inverse heat conduction problem (IHCP).

**Results:**

In the present study, these parameters were optimized using artificial neural networks in order to localize the heat source in the medium (i.e. female breast) more accurately.

**Conclusion:**

Eventually, a new formula was derived from Penne’s bioheat equation to estimate the depth and radius of the embedded heat source. Moreover, by analyzing the data, errors of the parameters could be estimated.

## Background

Breast cancer is the second leading cancer in the female population. According to the US breast cancer statistics, 1 in 8 women (over 12%) will develop breast cancer during their lifetime [1].

Detecting breast cancer at earlier stages will increase survival time (defined as the time between the date of diagnosis and the date of death)—unless survival time is shortened by (aggressive) treatment [1–4]. At present, there are many imaging techniques available for the detection of breast tumors, including magnetic resonance imaging (MRI), X-ray, positron emission tomography (PET), and mammography. A distinctive similarity among all these imaging methods is that they are either invasive or associated with ionizing radiation. Unlike the aforementioned approaches, infrared (IR) thermography can be used as a non-ionizing, non-contact, and safe method for detecting the location, size, and different thermal parameters of a tumor at early stages [3, 4].

Currently, infrared imaging being used in medicine for various applications that such as evaluation of allergic tests, morphea, basal cell carcinoma, chilblains, melanocytic naevi, melanoma extensivity, deep vein thrombosis, burn depth, Raynaud’s phenomenon, thyroid gland changes, pneumonia development, arthropathy and many other pathological conditions [3, 5]. Infrared imaging provides information about the temperature by measuring the radiation emitted from the skin surface. This technique was not well accepted in the past due to its high cost and the low sensitivity of IR cameras. However, advances in IR technology have promoted its application in medicine as a low-cost, non-invasive, and non-ionizing technique [3, 4].

Skin surface temperature can be affected by several parameters such as the blood perfusion rate underneath the skin, metabolism rate, and the heat exchange rate between the body surface and the surrounding environment. Any change in such parameters can change the body surface temperature, as well as heat flux on the skin surface [6–8]. Cancerous tissues frequently produce more heat than normal tissues due to their high vasculature (which may result in an altered localized thermal gradient due to heat conduction to neighbouring cooler surrounding) in addition to a higher metabolic rate (with rapidly dividing malignant cells). Therefore, the skin above such tissues will have a higher local temperature by several degrees. This abnormality in skin temperature can be utilized for detection of tumor parameters and evaluating the tumor at different stages of treatment [2–4, 6, 9–11]. With most of the present scanning techniques, an aggressive tumor which is <2 mm in width may remain undetected. Such small undetected tumors will grow, making the treatment more difficult and reducing the chance of survival [12–16].

Surface temperature for tumor detection was first employed by Lawson in 1959 [17]. In 1969, Draper and Jones used IR scans to examine the relationship between the body temperature and different diseases [18]. In 1971, Feasy et al. used point heat sources to investigate the effects of natural and forced cooling on tumors’ thermographic patterns [19]. The findings of this study suggest that tumor depth alone affects temperature distribution. Hence, surface cooling will not affect the temperature distribution [9, 19]. Also, in 1972, Davison et al. suggested that there exists a “linear relation between the depth of tumor and the width at half height of the resultant skin temperature distribution”, which is independent of the tumor radius [20]. During past decades, however, more studies have been conducted on this subject, among which was thermographing breast tumors in 5800 patients in order to measure the tumor temperature [9, 21].

A further aspect of IR imaging techniques and detection methods of breast cancer from IR images are described in detail by Ng in [22]. According to this paper, several conditions such as environmental temperature, temperature reference, and patient and camera positions should be taken into consideration before conducting the thermal imaging. Additionally, image capture protocol is considered to be the major source of error due to the camera focus distance and its viewing angles [22].

In this work, given the complexity of the human body and tumors, an algorithm has been developed under certain assumptions to estimate the location and depth of the tumor. Afterward, the results were optimized using an artificial neural network (ANN), an information processing algorithm that behaves similarly to the biological nervous system for processing information [2, 3, 23]. An ANN algorithm is composed of many interconnecting processing elements or neurons. In addition, it is employed for solving problems such as data processing and pattern recognition. The ANN learns by example and the initial parameters [23].

In the present study, the heat source problem was analyzed using two different approaches. First, the effects of the intensity, depth, and radius of the heat source on the skin surface temperature were analyzed. Afterward, the values of the parameters were optimized for a more accurate localization.

In the second part, the sensitivity of each parameter was studied. Moreover, an equation was derived which utilizes only the maximum temperature, and the local temperature (at any distance from the maximum temperature) on the skin surface in order to calculate the depth, and hence the radius, of the heat source without the need for measuring the heat source intensity.

## Methods

### Measurement estimation

As stated earlier, infrared thermography can be used for measurement of the skin surface temperature. The bioheat model was used in this study. To calculate tumor parameters from the obtained thermogram, it is essential to solve for the inverse bioheat equation [24, 25]. This model was developed by solving the inverse heat conduction problem (IHCP) based on Penne’s bioheat transfer equation [10, 26]:

1 $$\begin{aligned} \uprho {\text{c}}\left( {{{\partial {\text{T}}\left( {\overrightarrow {\text{r}} , {\text{t}}} \right)} \mathord{\left/ {\vphantom {{\partial {\text{T}}\left( {\overrightarrow {\text{r}} , {\text{t}}} \right)} {\partial {\text{t}}}}} \right. \kern-0pt} {\partial {\text{t}}}}} \right) &= \nabla {\text{k}}\left( {\text{r}} \right)\nabla {\text{T}}\left( {\overrightarrow {\text{r}} , {\text{t}}} \right)+ \upomega_{\text{b}} \uprho_{\text{b}} {\text{c}}_{\text{b}} \left[ {{\text{T}}_{\text{a}} \left( {\overrightarrow {\text{r}} , {\text{t}}} \right) - {\text{T}}\left( {\overrightarrow {\text{r}} , {\text{t}}} \right)} \right] \\ & \quad + {\text{Q}}\left( {\overrightarrow {\text{r}} , {\text{t}}} \right) \to \overrightarrow {\text{r}} \in {\text{Q}} \end{aligned}$$

In this equation, $\uprho {\text{c}}\left( {{{\partial {\text{T}}\left( {\overrightarrow {\text{r}} , {\text{t}}} \right)} \mathord{\left/ {\vphantom {{\partial {\text{T}}\left( {\overrightarrow {\text{r}} , {\text{t}}} \right)} {\partial {\text{t}}}}} \right. \kern-0pt} {\partial {\text{t}}}}} \right)$ is a transient term indicating the change in the temperature as a function of time. $\nabla {\text{k}}\left( {\overrightarrow {\text{r}} } \right)\nabla {\text{T}}\left( {\overrightarrow {\text{r}} , {\text{t}}} \right)$ is the heat conduction equivalent. Additionally, ρ and c are defined as the density and specific heat of the tissue. k(r) is the thermal conductivity of the medium. $\upomega_{\text{b}} \uprho_{\text{b}} {\text{c}}_{\text{b}} \left[ {{\text{T}}_{\text{a}} \left( {\overrightarrow {\text{r}} , {\text{t}}} \right) - {\text{T}}\left( {\overrightarrow {\text{r}} , {\text{t}}} \right)} \right]$ defines blood perfusion and ${\text{Q}}\left( {\overrightarrow {\text{r}} , {\text{t}}} \right)$ is the metabolic heat rate [10, 26].

For a feasible solution for the IHCP, several assumptions have been made [10].

1. Solving the equation for breast tissue, which is mainly composed of adipose tissue.
2. Imaging the surface temperature of the model using an IR camera. The generated bioheat model is assumed to be static, and hence time has no effect on the model.
3. The model is homogeneous and isotropic with constant ρ, c, and k.
4. $\upomega_{\text{b}} \uprho_{\text{b}} {\text{c}}_{\text{b}} \left[ {{\text{T}}_{\text{a}} \left( {\overrightarrow {\text{r}} , {\text{t}}} \right) - {\text{T}}\left( {\text{r,t}} \right)} \right]$ and ${\text{Q}}\left( {\overrightarrow {\text{r}} , {\text{t}}} \right)$ are considered to be a constant heat source embedded in breast tissue. The heat generated by the tumor is assumed as an additional heat source. These two were addressed by solving for an “equivalent point source” problem.

Hence, Eq. (1) can be rewritten as:

2 $$\uprho {\text{c}}\left( {\partial {\text{T}}\left( {{{\left( {{\vec{\text{r}}}} \right)} \mathord{\left/ {\vphantom {{\left( {{\vec{\text{r}}}} \right)} {\partial {\text{T}}}}} \right. \kern-0pt} {\partial {\text{t}}}}} \right)} \right) = \nabla \left[ {{\text{k}}\nabla {\text{T}}\left( {{\vec{\text{r}}}} \right)} \right] + {\text{Q}}\updelta \left( {{\vec{\text{r}}}} \right)$$

with ∂T/∂t = 0 since the model is static.

By further reducing Eq. (2), we obtain [10]:

3 $$\nabla^{2} {\text{T}}\left( {\overrightarrow {\text{r}} } \right) = \left( {{{ - 1} \mathord{\left/ {\vphantom {{ - 1} {\text{k}}}} \right. \kern-0pt} {\text{k}}}} \right){\text{Q}}\updelta \left( {\overrightarrow {\text{r}} } \right)$$

where ∇^2^ = (∂^2^/∂x^2^) + (∂^2^/∂y^2^) + (∂^2^/∂z^2^) is the Laplace term.

At the tissue surface, heat transfer is mainly a convection mechanism [10].

In order to solve for a three-dimensional model, a spherical coordinate was developed with the point heat source at the origin with body temperature as a function of r, φ, and θ. Also, there exists a spherical symmetry with $\frac{\partial T}{\partial \theta } = 0$ and $\frac{\partial T}{\partial \varphi } = 0$ [1, 2, 9].

4 $$\frac{1}{{r^{2} }}\frac{\partial }{\partial r}\left( {r^{2} \frac{\partial T}{\partial r}} \right) + \frac{1}{{r^{2} sin\theta }}\frac{\partial }{\partial \theta }\left( {sin\theta \frac{\partial T}{\partial \theta }} \right) + \frac{1}{{r^{2} sin\theta }}\frac{{\partial^{2} T}}{{\partial \varphi^{2} }} = \frac{ - Q}{k}\delta (r)$$

5 $$\frac{1}{{r^{2} }}\frac{\partial }{{\partial r}}\left( {r^{2} \frac{{\partial T}}{{\partial r}}} \right) = \frac{{ - Q}}{k}\delta (r)$$

**Fig. 1:**
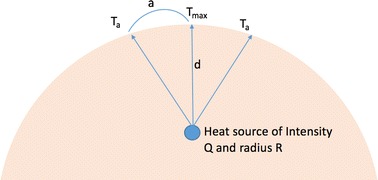
Depth, intensity, and radius measurement model [2, 3]

This problem is solved in two different steps (as illustrated in Fig. 1, next page) by assuming [9]:

1. r ≠ 0
2. r = 0

For the first assumption, Eq. (5) transforms into [9, 10]:

6 $$\frac{{\partial T^{2} }}{{\partial r^{2} }} + \frac{2}{r}.\frac{\partial T}{\partial r} = 0$$

with a general equation form of T = (−c/r) + D with a constant c and D (for environment temperature).

In addition, for the latter assumption, r = 0, the volume integral is used to solve for the problem, with r being the radius of the sphere (a positive number ε) and v being the sphere itself. Therefore, Eq. (6) becomes [10]:

7 $$\iiint {\nabla^{2} {\text{T}} . {\text{dv}}_{\upvarepsilon } = - {\text{Q}}/{\text{k }}\smallint \smallint \smallint \updelta \left( {\text{r}} \right).{\text{ dv}}_{\upvarepsilon } }$$

According to the Gaussian theorem:

8 $$\smallint \smallint \smallint \nabla^{ 2} {\text{T}}.{\text{dv}}_{\upvarepsilon } = {\mathop{{\int\!\!\!\!\!\int}\mkern-21mu \bigcirc} }\nabla {\text{T}}.{\text{ds}}$$

Then function Eq. (5) becomes:

9 $$\mathop{{\int\!\!\!\!\!\int}\mkern-21mu \bigcirc} {\frac{c}{{r^{2} }}} .{\text{ds = }}{{ - {\text{Q}}} \mathord{\left/ {\vphantom {{ - {\text{Q}}} {\text{k}}}} \right. \kern-0pt} {\text{k}}}$$

To find C we can take the limit of Eq. (9):

10 $${\lim}_{\varepsilon \to 0} {\mathop{{\int\!\!\!\!\!\int}\mkern-21mu \bigcirc} }\frac{C}{{r^{2} }}.{\text{ds }} = \frac{C}{{\varepsilon^{2} }}.4\pi \varepsilon^{2}$$

11 $${\text{C}} = \frac{ - Q}{4\pi k}$$

Also, assuming the environmental temperature to be T0:

12 $${\text{D}}\,{ = }\,{ \lim }_{\varepsilon \to \infty } T = {\text{T0}}$$

Equation (12) is the mathematical representation of environment temperature. It describes the fact that environment temperature stays constant and does not depend to the size of tumor.

Finally, the temperature distribution of the tissue in three-dimensional coordinates can be expressed as [10]:

13 $$T\left( {r,\varphi ,\theta } \right) = \frac{Q}{4\pi kr} + {\text{T0}}$$

Equation (13) is the three-dimensional representation of temperature distribution. As it is mentioned earlier in this paper there exists a spherical symmetry with $\frac{\partial T}{\partial \theta } = 0$ and $\frac{\partial T}{\partial \varphi } = 0$, therefore, the temperature is only distributing as a function of the radius of the tumor. Hence $\frac{1}{{r^{2} }}\frac{\partial }{\partial r}\left( {r^{2} \frac{\partial T}{\partial r}} \right) = \frac{ - Q}{k}\delta (r).$

A cancerous tissue can be modeled with intensity Q, radius R, and depth d in spherical coordinates. Considering an ideal condition in which the heat radiating from a heat source is completely exchanged with the surrounding environment [2, 3]:

14 $$Q_{in} = Q_{out}$$

Combining Eqs. (13) and (14), we obtain [2]:

15 $${{\text{Q}} \mathord{\left/ {\vphantom {{\text{Q}} 4}} \right. \kern-0pt} 4}\left[ {\left( {{\text{d}} + {\text{R}}} \right)^{ 2} + {\text{a}}^{ 2} } \right] = {\text{h}}0 \, \left( {{\text{T}}_{{({\text{a}})}} - {\text{T}}_{\text{e}} } \right)$$

Hence [2]:

16 $${\text{T}}_{{({\text{a}})}} = {\text{T}}_{\text{e}} + \, {{\text{Q}} \mathord{\left/ {\vphantom {{\text{Q}} {\left( { 4 {\text{h}}_{0} \left[ {\left( {{\text{d}}\,{ + }\,{\text{R}}} \right)^{2} + {\text{a}}^{2} } \right]} \right)}}} \right. \kern-0pt} {\left( { 4 {\text{h}}_{0} \left[ {\left( {{\text{d}}\,{ + }\,{\text{R}}} \right)^{2} + {\text{a}}^{2} } \right]} \right)}}$$

where *h*_0_ is the heat exchange coefficient, T_(a)_ is the temperature at the surface, and T_e_ is the environment temperature [3].

The heat source parameters such as depth, radius, and heat intensity of the proposed model are calculated through the following equations [3]:

17 $$d = \frac{{a\sqrt {{\text{T}}({\text{a}})-Te} }}{{\sqrt {T_{{max}} -T(a)} }}$$

18 $${\text{Q}}{\mkern1mu} ={\mkern1mu}4\pi h0\frac{{(T\left( a \right) - Te){\mkern1mu}(T_{{max}}-Te)}}{{T_{{max}} -T(a)}}a^{2}$$

19 $${\text{R = }}\sqrt[3]{{\frac{Q}{{Q_{m} A_{t} }}}}$$

where Q_m_ is the metabolic heat generation (W/m^3^), and A_t_ is the volume of a single cell as 1 μm [2, 3].

The heat source parameters are illustrated in Fig. 1.

Since the thermal emissivity of the skin surface varies with the angle at which it is viewed, for more accurate reading, the tissue is viewed from the top.

## Results and application

Equation (16) was used to assess the effects of the d, Q, and R parameters on the model.

The values of the thermal and physical properties of sound breast tissue are listed in Table 1 [4].

**Table 1:** Thermal and biological parameters

Parameter	Symbol	Value	Unit
Thermal conductivity	k	0.52	W/(m K)
Heat exchange coefficient	h_a_	8.77	W/(m^2^ K)
Specific heat (blood)	c_b_	4186	J/(kg K)
Density (blood)	ρ_b_	1000	kg/m^3^
Metabolic heat generation (breast)	q_m_	700	W/m^3^
Metabolic heat generation of tumor	Q_t_	25,000–90,000	W/m^3^
Perfusion rate	ω_b_	0.00052	1/s
Arterial blood temperature	T_a_	310.15	K
Environmental temperature	T_e_	300.15	K

### Effect of the heat source parameters on surface temperature

To assess the effect of the heat source parameters on the proposed model, a parametric study was performed by varying one parameter at a time while keeping the other two constant. The analytic method was developed using MATLAB R_2015b through out this research.

First, the model was simulated by applying a constant heat source intensity of 0.1 W and changing the depth of the heater embedded in the model. The heat source was assumed as a point, hence R = 0.

**Fig. 2:**
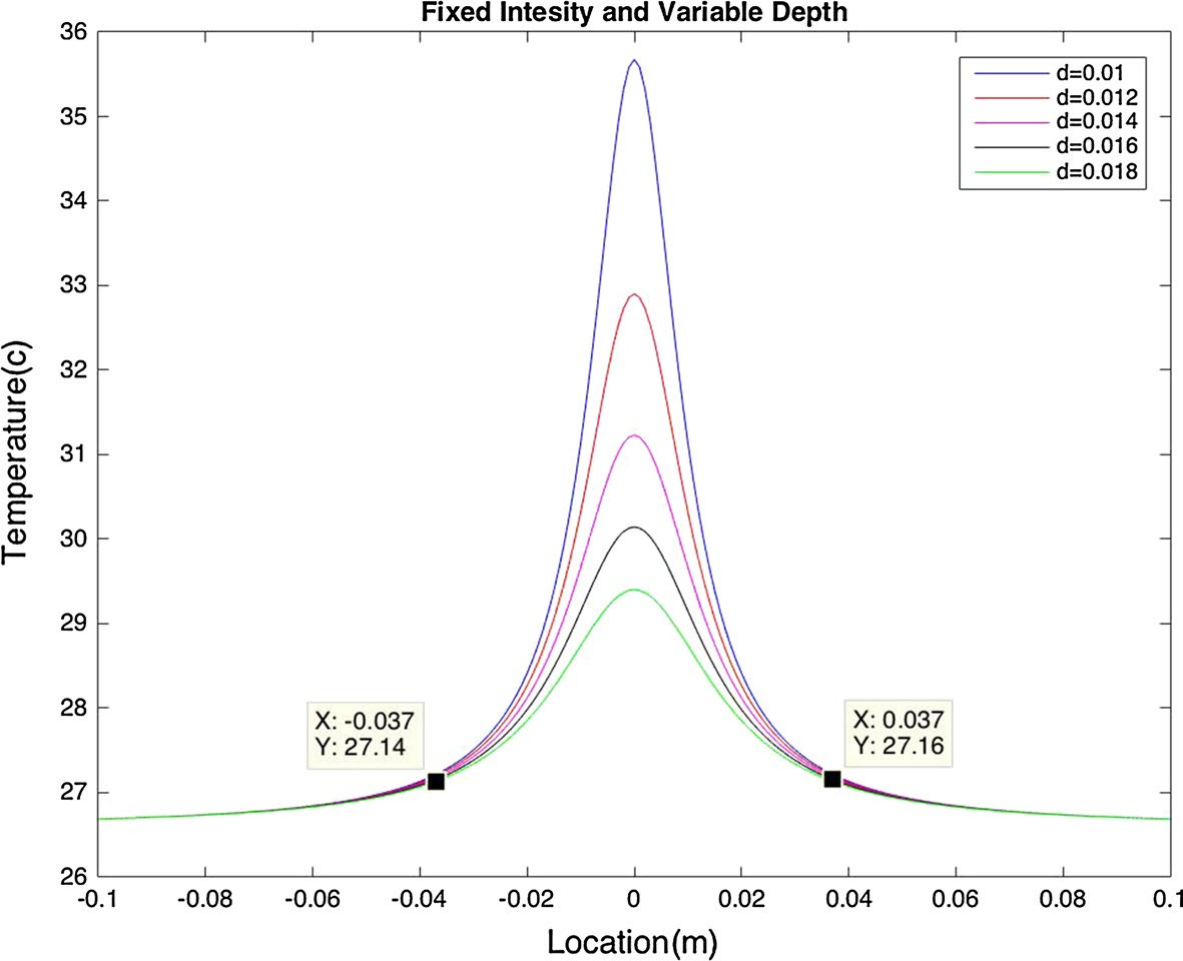
Fixed intensity and varying depth: surface temperature directly depends on the depth at which the heat source is located

The depth of the heat source affects the maximum temperature (T_max_) as shown in Fig. 2. However, the area of temperature elevation is not affected by varying the depth. It can also be observed that the temperature rises significantly when the heat source is located (depth) around ±0.037 m.

Next, the model was used by positioning the heat source at the depth of d = 0.014 m and varying the intensity. The radius was zero.

**Fig. 3:**
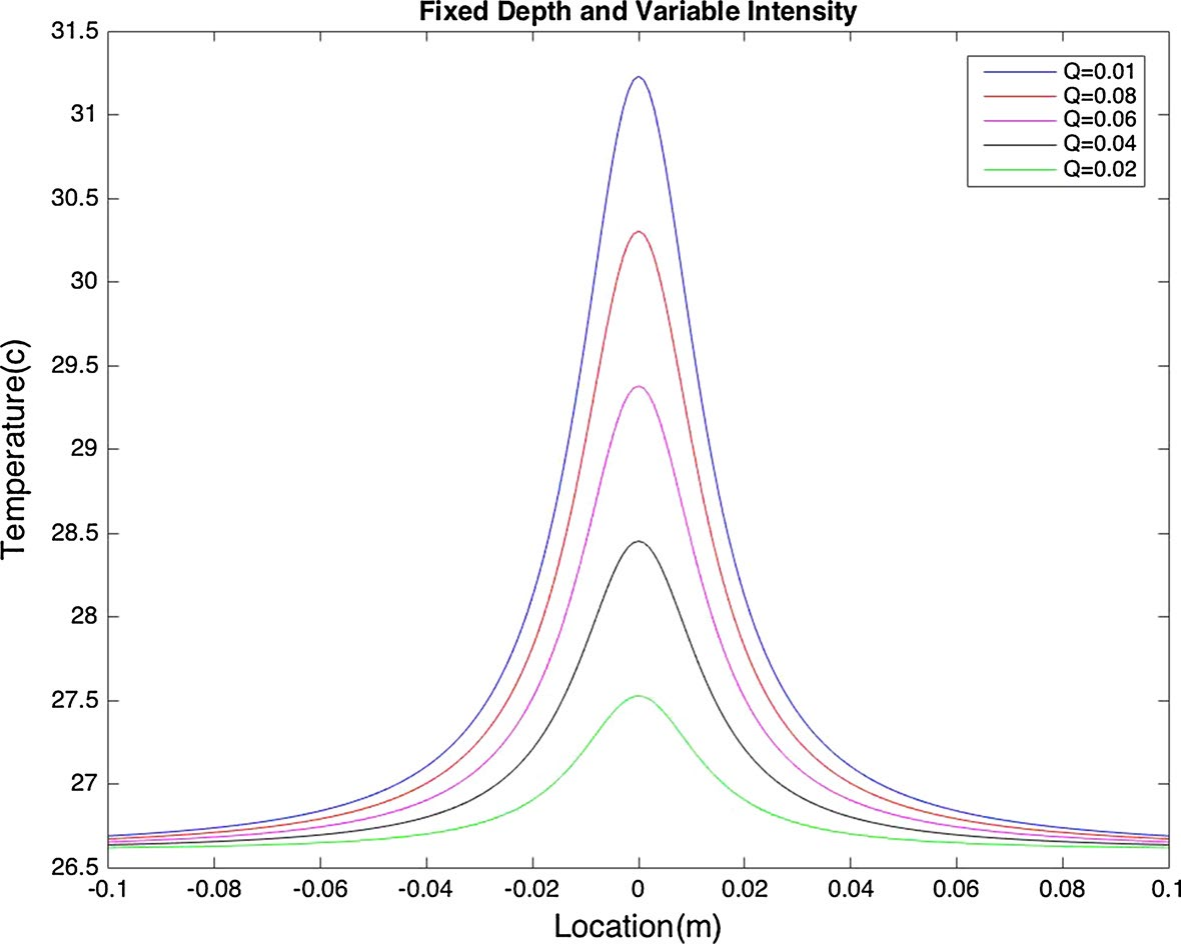
Fixed heat source depth and varying intensity: variation in intensity affects the area over which temperature rises

Variation in the intensity affects both the maximum temperature and the area over which the temperature rises, as illustrated in Fig. 3.

**Fig. 4:**
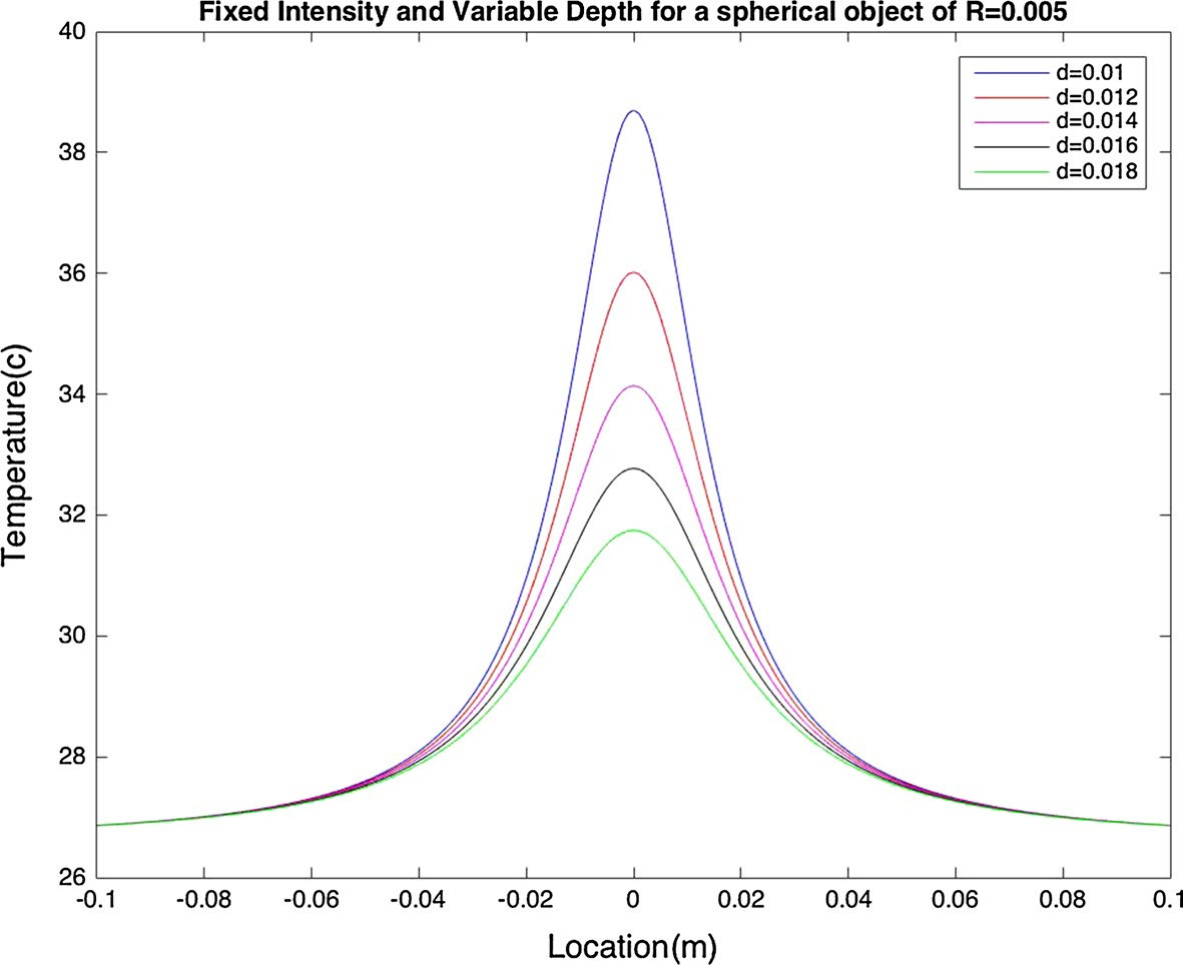
Fixed intensity and varying depth for a spherical object with R = 0.005: temperature on the model surface directly depends on the depth at which the heat source is located

**Fig. 5:**
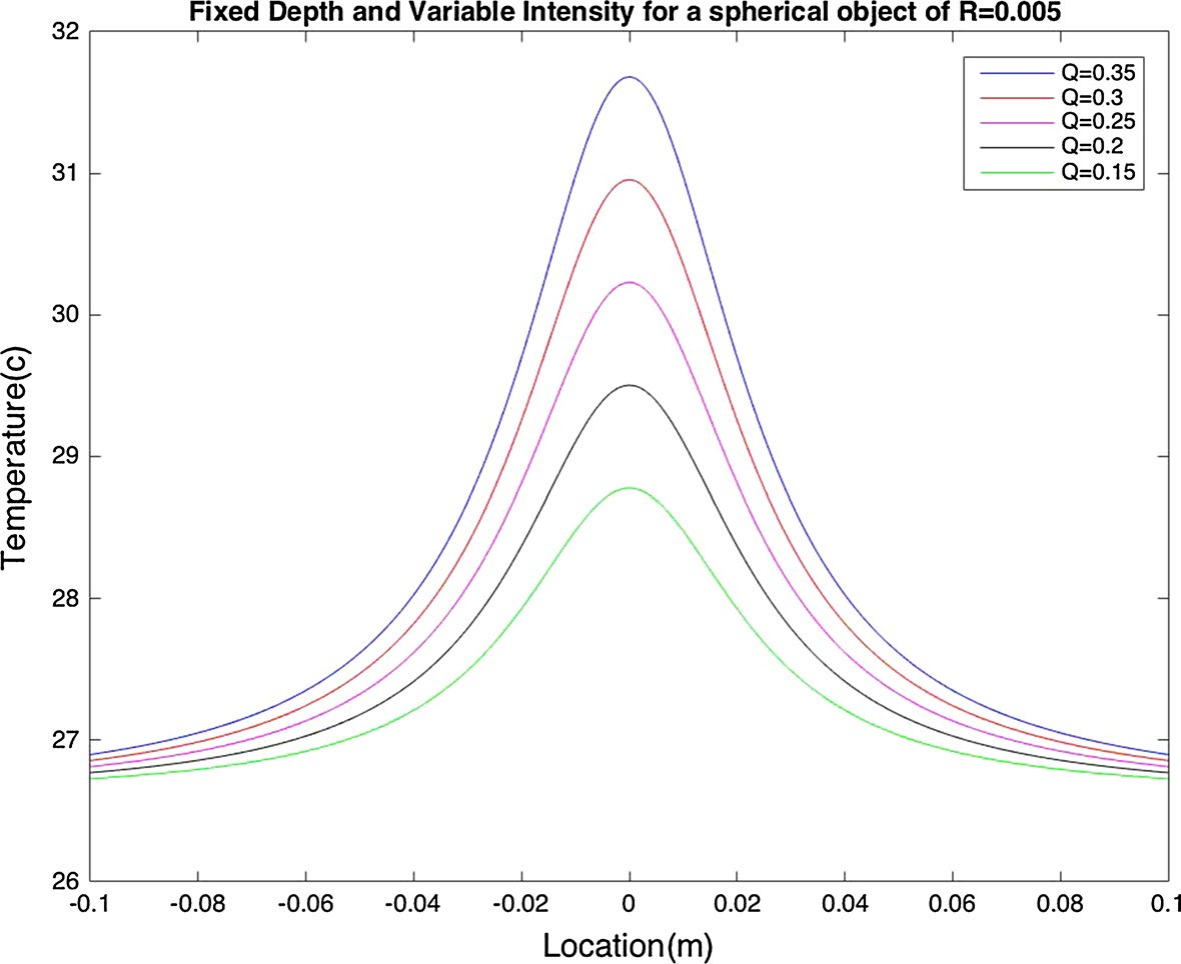
Fixed heat source depth and varying intensity for a spherical object with R = 0.005: variation in intensity affects the area over which temperature rises

In addition, a condition in which the heat source is not a point, i.e. R ≠ 0, was assumed. In Fig. 4, the heat source radius and intensity were assumed to be 0.005 m and 0.3 W, respectively. In Fig. 5, however, the heat source depth was fixed at d = 0.02 m, while R = 0.005.

It can be seen that a model with a spherical heat source of radius R has similar effects on the surface temperature to the point heat source with R = 0 (R is a very small number in this case so it is possible to ignore its value). As illustrated in Fig. 4, variation in the depth affects T_max_. Also, the area over which the temperature changes is affected by varying intensity, as can be seen in Fig. 5.

**Fig. 6:**
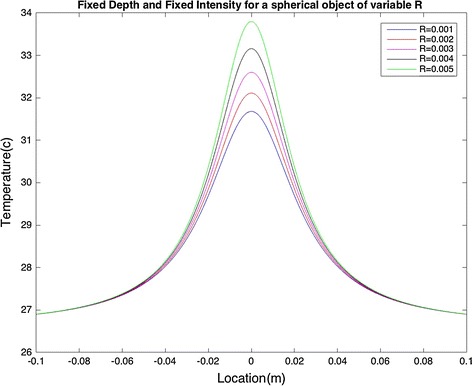
Fixed heat source depth and fix intensity for spherical objects with varying Rs

Figure 6 shows the effect of radius variation (R) on the surface temperature, for the depth of heat source and intensity of d = 0.02 m and Q = 0.3 W, respectively. It can be observed that as the radius increases, the maximum temperature increases as well. Furthermore, the area over which the temperature rises is unchanged. However, by comparing Fig. 6 with Figs. 4 and 5, we can conclude that in comparison with changes in the depth and intensity, changes in the radius do not have a significant effect on the surface temperature.

### ANN model

The depth, radius, and intensity of the model, found through the model above, were optimized by training the artificial neural network via (ANN) toolbox of MATLAB. A 3-layer feed-forward ANN with back propagation learning was developed to represent the surface temperature. The model was simulated using various parameters and the results were used for training the ANN. The algorithm was developed to have 3 inputs, and 10 hidden layers.

**Fig. 7:**
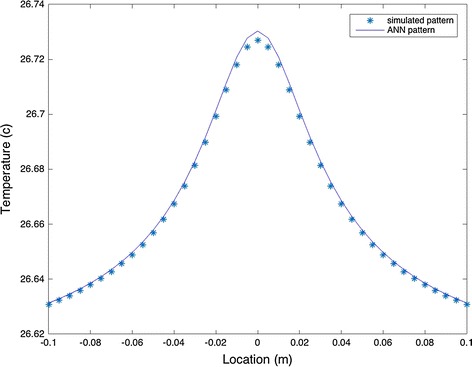
Heat source model and ANN simulated model

The ANN was trained for two sets of data. The first dataset was obtained assuming the ideal (noiseless) situation. Using Eqs. (18) and (19), the parameters of the model were found to be Q = 0.35 W, d = 2 cm, and R = 5 mm. Also, the initial values were assumed to be Q = 0.356 W, d = 2.02 cm, and R = 5 mm. Using the initial values to train the ANN, these parameters were optimized to Q = 0.3559 W, d = 2.03 cm, R = 4.5 mm. Comparing the maximum temperature of the model with the maximum temperature obtained from ANN, it could be seen that there is 0.01 °C difference (Fig. 7). However, the difference is negligible due to the low intensity of the tumor. As the intensity increases, the difference between the two maximum temperatures increases too. Assuming Q = 5 W, d = 1 cm, and R = 5 mm and training the ANN with initial values of Q = 5.5 W, d = 3 cm, and R = 0 mm, the new optimized values for these parameters will be Q = 5.5276 W, d = 1.62 cm, and.

R = 4.1 mm with the maximum temperature difference of 0.3611 °C. Based on our previous observations (see Figs. 3, 5), these results were expected.

For the second dataset, a random noise of 10% was added to the model. This noise could be in form of ambient temperature, Therefore, the parameter values were changed to Q = 0.05 W, with d = 1 cm, and R = 0.09 mm. By simulating and training the ANN, the optimized values of Q = 0.0478 W, d = 0.0296 m, and R = 0.1 mm were obtained. In this case, the maximum temperature difference was 0.03 °C.

**Fig. 8:**
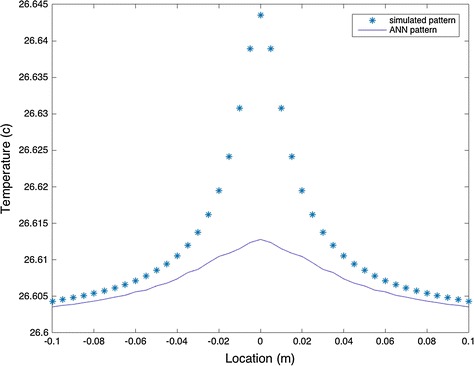
Noisy model of analytical solution with and without ANN simulation. Maximum temperature increases after optimization process to a more accurate temperature

**Fig. 9:**
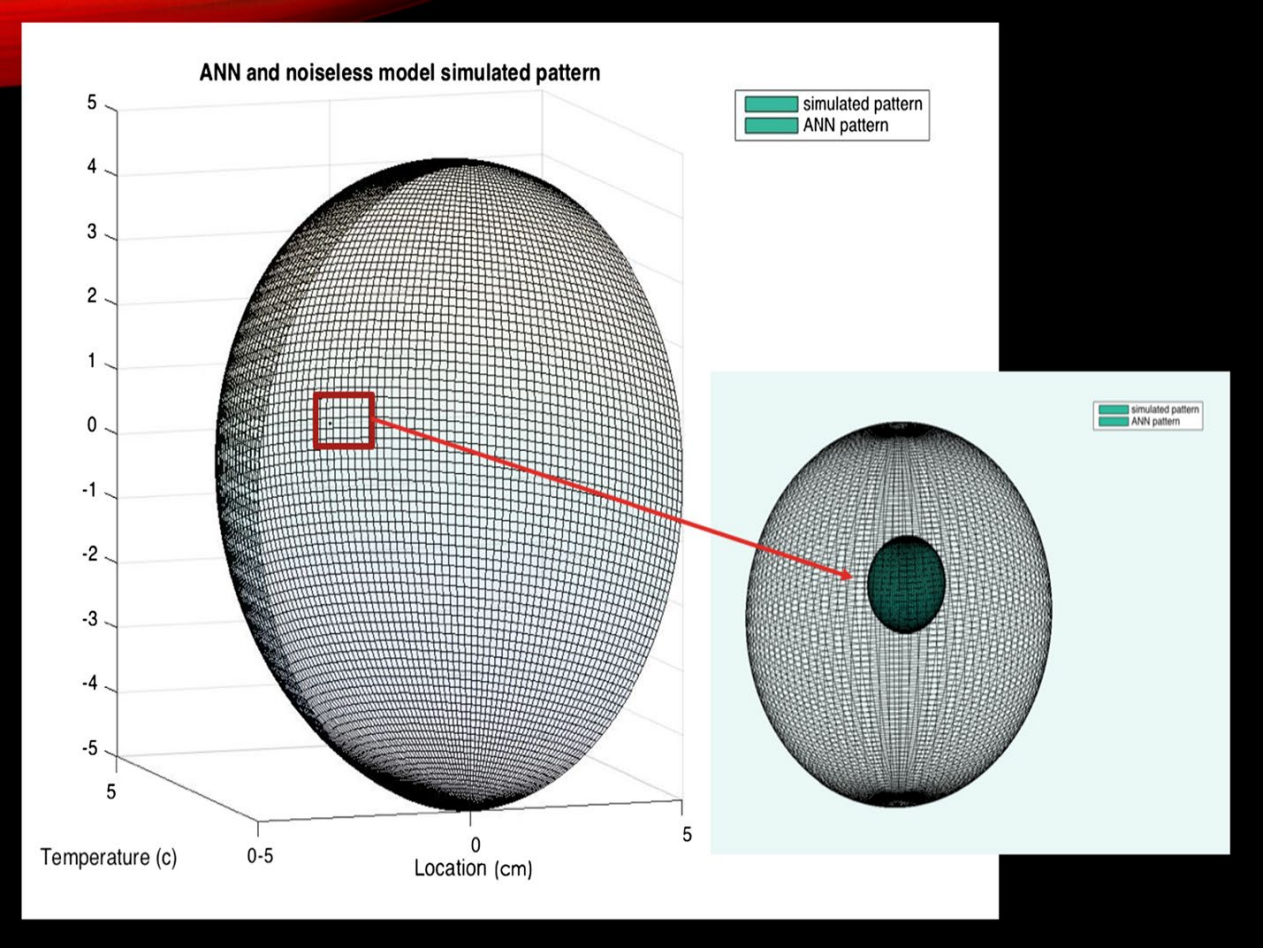
A comparison between the optimized and non-optimized parameters (depth and radius) assuming a spherical breast of diameter 10 cm. The larger sphere illustrates the optimized location of the tumor within the breast tissue. The hemisphere outside the red box represents the breast tissue

As Figs. 7 and 8 show, there is only a maximum of 1.66 and 4.4% error in the value of Q for datasets I and II, respectively. Figure 9 compares the optimized parameters with the non-optimized ones. Using the parameters before optimization, the size and location of the tumor is much smaller that running the simulation with optimized values.

Table 2 illustrates the error analysis for the noisy and no noise conditions. As it could be concluded, the higher the intensity, the higher the error in parameter estimation. This error would be reflected as temperature difference on the skin surface for various ranges and would affect the correct estimation of tumor location. Therefore, its critical to use the optimized model for research and clinical perpuses.

**Table 2:** Error analysis for optimization of the two cases

Parameter	Original value	Optimized value	Error (%)
Data set (I): A condition with no noise and low intensity of tumor			
Depth (d)	2 cm	2.03 cm	1.5
Raduse (R)	5 mm	4.5 mm	10
Intensity (Q)	0.37 W	0.3559 W	3.81
Data set (I): A condition with no noise and higher intensity of tumor			
Depth (d)	1 cm	1.62 cm	62
Raduse (R)	5 mm	4.1 mm	18
Intensity (Q)	5 W	5.5276 W	10.55
Data set (II): A condition with 10% noise			
Depth (d)	1 cm	2.96 cm	66.2
Raduse (R)	0.09 mm	0.1 mm	11.11
Intensity (Q)	0.05 W	0.0478 W	4.4

### Sensitivity of the data analysis

#### Parameter sensitivity

**Fig. 10:**
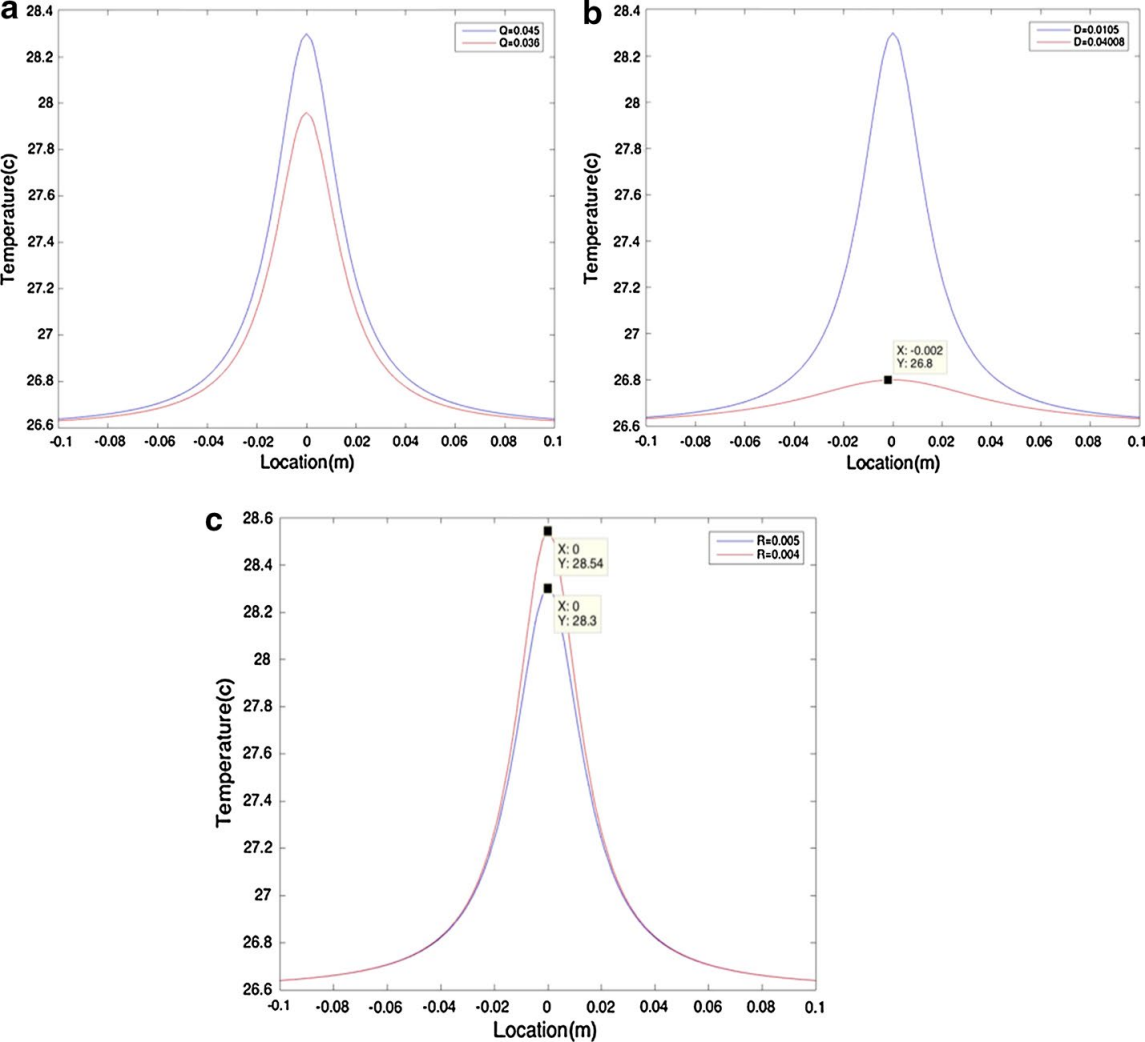
Sensitivity studies with 20% change in data. **a** illustrationg difference of 0.3 °C between the initial Q and Q measured. **b** illustrating 1.5003 °C as the depth of the heat source was increased by 20%. **c** changing R by 20% was accompanied by a temperature difference of 0.24 °C

To analyze the sensitivity of each parameter, a model was developed with the following parameters: Q = 0.045 W, d = 1.05 cm, and R = 5 mm. Subsequently, these parameters were changed by ±20%. In the first step, all parameters were kept constant, and only the intensity was adjusted to Q = 0.036 W. As shown in Fig. 10a there was only a small difference of 0.3 °C between the initial Q and Q measured with 20% error. However, the difference increased to about 1.5003 °C as the depth of the heat source was increased by 20% (Fig. 10b). Additionally, changing R by 20% was accompanied by a temperature difference of 0.24 °C (Fig. 10c). Therefore, it could be concluded that these parameters are mostly sensitive to changes in depth, since a small change in depth resulted in over 1 °C change in temperature difference.

Considering a case with a heat source of higher Q, R, and D and applying the same ±20%, the generated graphs would follow the same trend, as illustrated in Fig. 10.

Additionally, we have allowed Q = 0.025 W and Q = 0.09 W. Adding 20% error to both we would have Q = 0.03 W and Q = 0.108 W. This eventually would result in temperature difference of 0.41 and 1.49 °C respectively.

#### Temperature sensitivity

A temperature sensitivity study was performed by assuming that there only exists information about the maximum temperature and two local temperatures, as shown in Fig. 1. Using geometric rules, the depth of the heat source was found. Subsequently, it was possible to find the heat source radius and precise location. The following equation was derived based on the bioheat model, showing the relationship between the depth of the heat source and its radius.

20 $$R^{3} = \frac{{\left( {T_{a} - T_{e} } \right)\left( {a^{2} + d^{2} } \right)4\pi h_{0} }}{{q_{m} \times 10^{ - 6} }}$$

Using this equation, it is possible to find the radius of the heat source without using the intensity. Equation (20) was obtained by solving the bioheat model. Values for *h*_0_ and q_m_ for breast tissue are listed in Table 1.

It would be more efficient to use Eq. (20) during biological and clinical testing, where the local and maximum temperatures are measured using an IR camera, rather than using the analytical solution, where the parameters of the tumor are found by solving analytical governing equations.

**Fig. 11:**
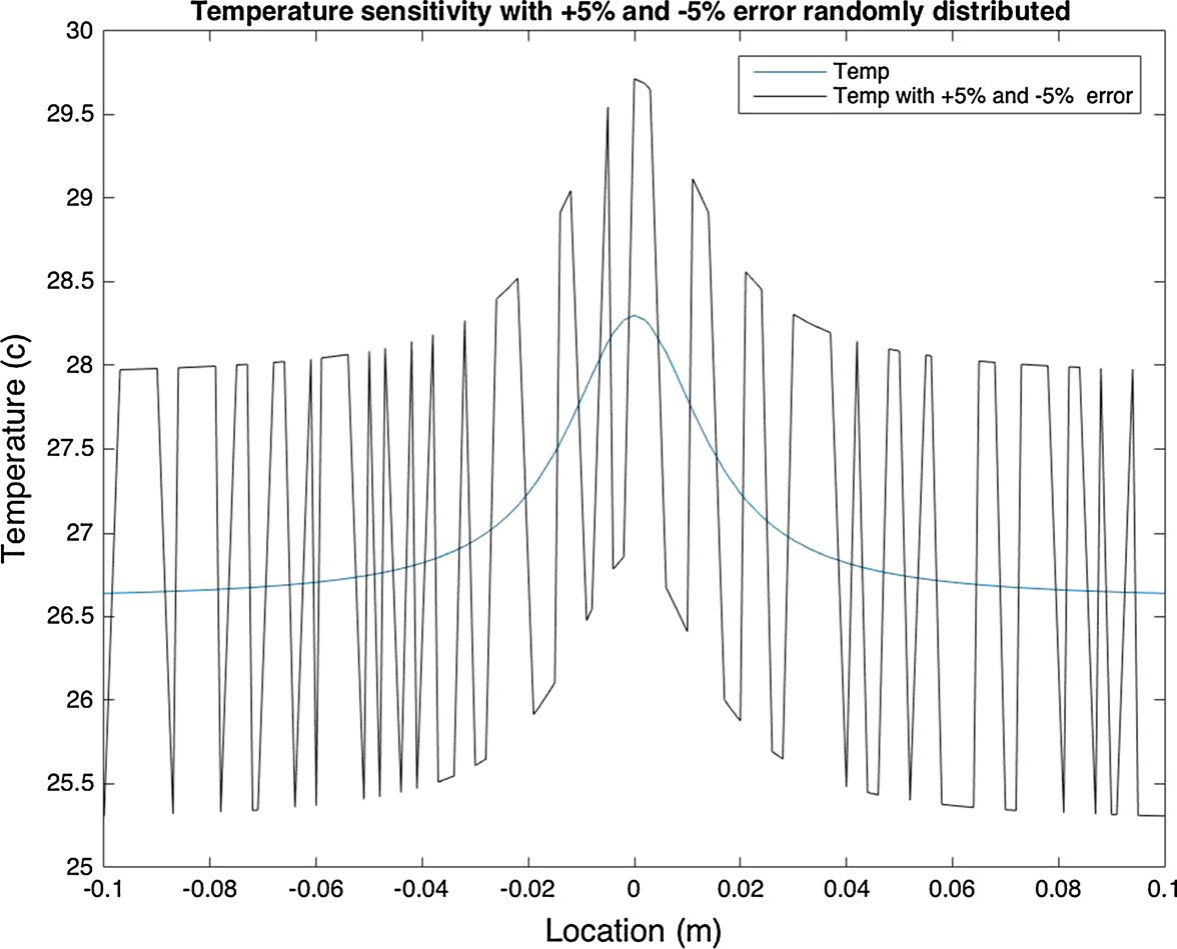
Sensitivity models with ±5% change in surface temperature. There is about 1.41 °C difference between maximum temperatures

The temperature sensitivity was studied assuming two conditions. The first condition was implemented by adding a random error of ±5% to the temperature at various locations. There is approximately 1.41 °C difference between the maximum temperatures, as shown in Fig. 11.

**Fig. 12:**
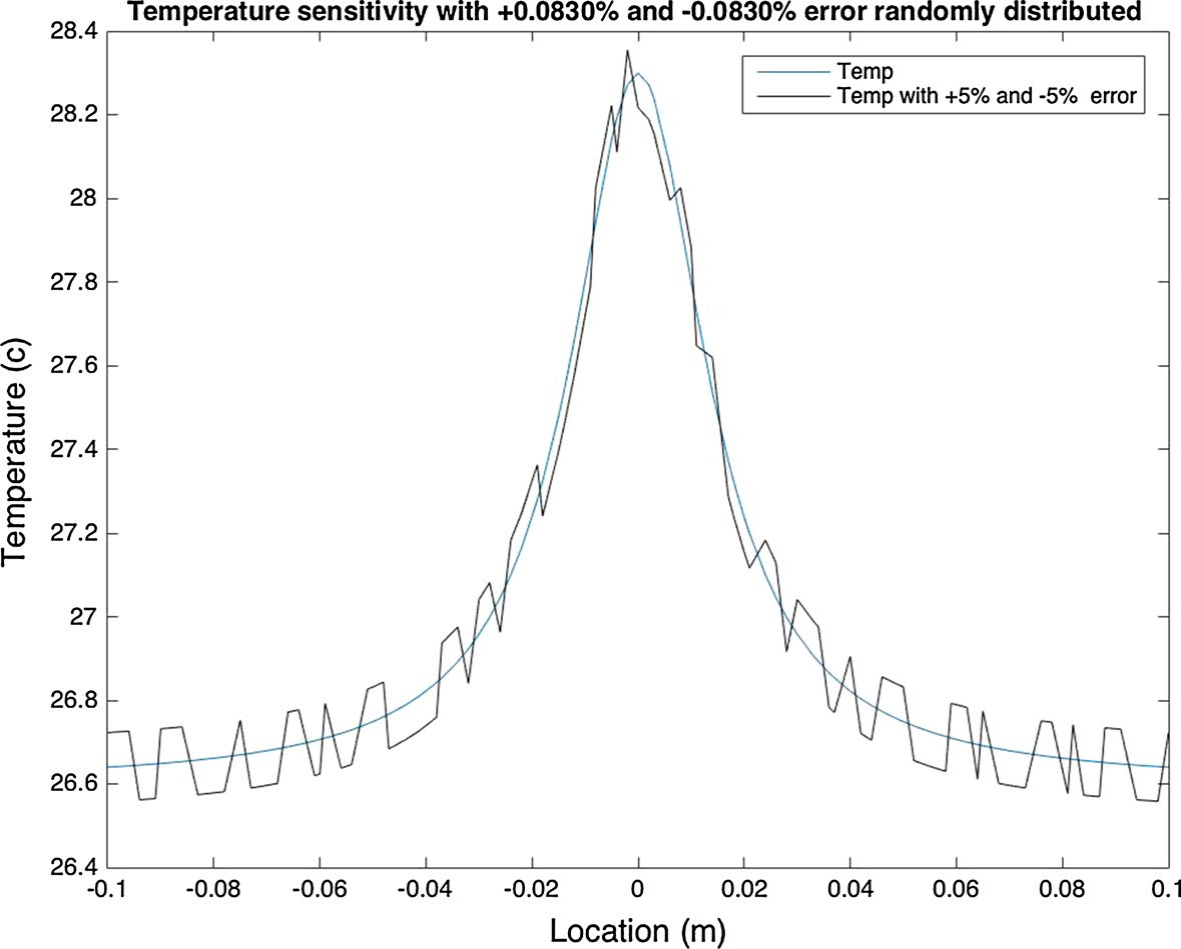
Sensitivity models with ±5% (of maximum and minimum points) change in surface temperature

In the second condition, the difference between the two temperatures decreases. Under this condition, the maximum and minimum temperatures were found to be 28.2996 and 26.6399 °C, respectively. Subtracting the two, there is 1.6597 °C temperature difference between the two extreme points. This difference was used to find the new value of temperature at each location, with a random ±5% error. Figure 12 illustrates the difference between the two maximum temperatures. This difference was around 0.0504 °C.

Comparing Figs. 11 and 12, it can be concluded that the surface temperature is extremely sensitive to any source of error. Therefore, it is vital to use a well calibrated camera in biological and clinical testings. This could be done by measuring the temperature of the object using a thermocouple and comparing it to the temperature obtained by the camera. It is recommended that in analytical research where an IR camera is not used, the heat intensity generated by the tumor should be measured first, and all other parameters should be calculated based on the intensity.

#### Image resolution

During image acquisition, the important factor is the focus of the camera. Given the camera is not in focus, the obtained temperature will be lower than the actual temperature of the surface causing a great error in tumor localization.

## Application

To apply this method, there is a need for three parameters namely room temperature, patient body surface temperature, as well as maximum temperature of any point on the breast. Room temperature can be measured by any digital thermometer. Maximum and body temperature can be measured via IR camera.

**Fig. 13:**
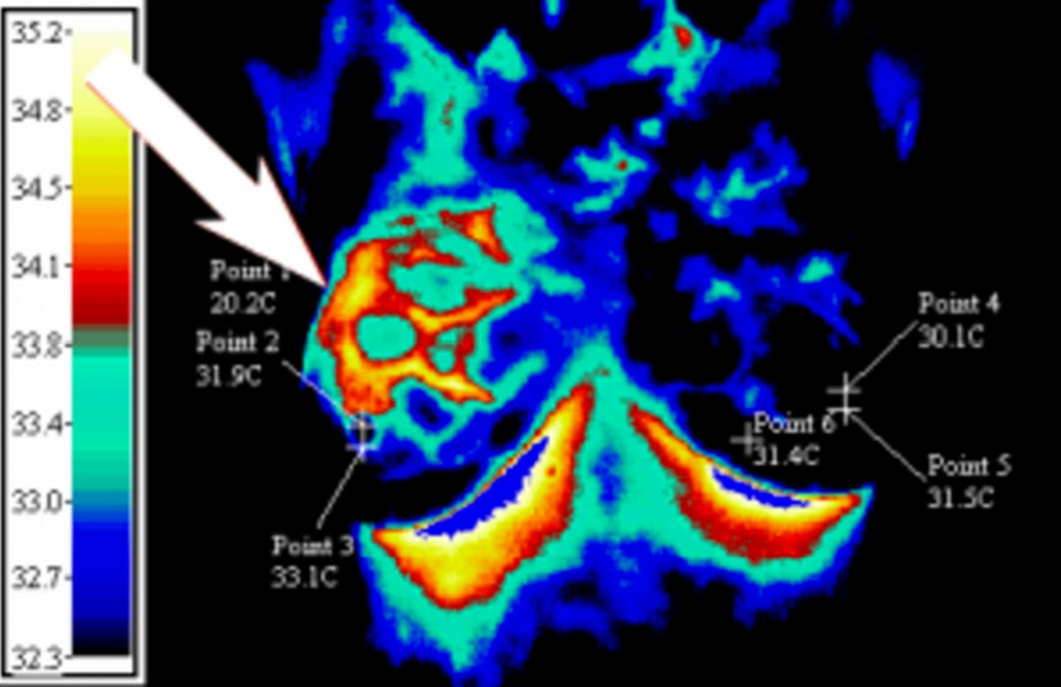
Thermal image of breast with an abnormal mass in the right breast

We have taken the Fig. 13 from clinicalthermography.com in order to carry out analysis using the proposed algorithm. Thermo graphic examinations were performed in a controlled 20 °C ambient temperature with normal skin temperature of 30 °C according to [21].

Additionally, according to [21], “Clinical experiments by Draper, indicate that only heat brought to within 6 mm (~¼′′) of the surface of the dermis is emitted.” Therefore, we ran the algorithm for tumors of 6 mm to 1 cm depth. We have used Eqs. (15) and (16) assuming the tumor has almost negligible radius (0.001 m).

Equation (15) was used to find Q using the parameters that were given (Ta = 33.4–35.1 °C and T_max_ = 35.2 °C according to Fig. 13). This equation provides us with a range for intensity, depending on the location the tumor that is found under the skin. We ran the algorithm multiple times and took the average of the results. This led to an intensity of 0.083204 W. Afterwards, we utilized Eq. (16) to find the surface temperature range, where Te = 20 °C, R = 0.001, a = −0.1:0.0005:0.1 m (from each side of the maximum temperature location), and T_max_ = 35.2 °C (according to Fig. 13).

**Fig. 14:**
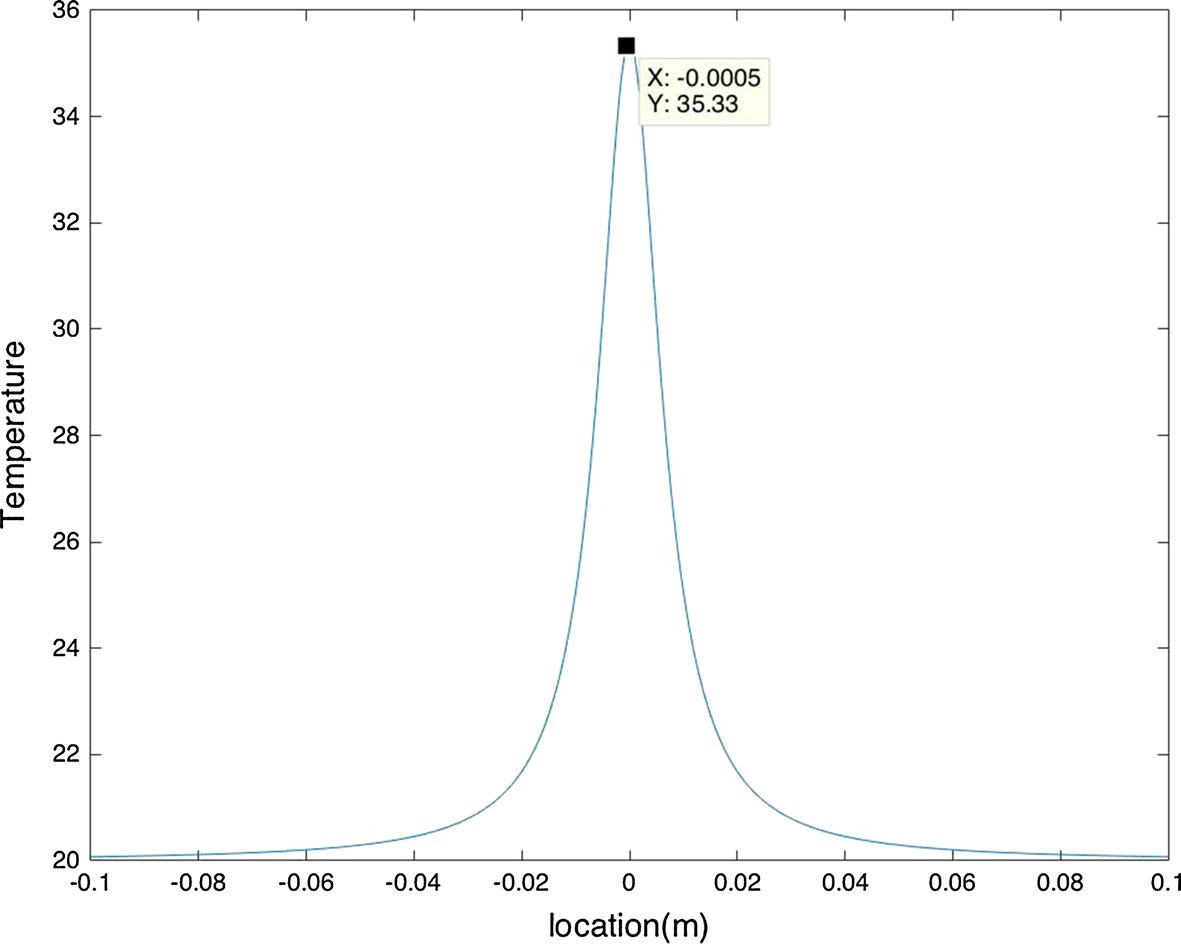
Maximum temperature provided by the algorithm

By using these parameters in the proposed algorithm, the local temperature (T) on the skin around the tumor were obtained as shown in Fig. 14.

As it can be seen from Fig. 14 the maximum temperature obtained by the algorithm is in good agreement with the one provided by Fig. 13.

## Conclusion

A methodology has been developed for the estimation of the thermo-physio-biological parameters of tumors using the temperature profile over the skin surface that has been obtained by solving bio-thermal problems on the proposed breast model. The proposed methodology was based on solving bio-thermal problems in the presence of a tumor. An artificial neural network (ANN) was used to optimize the tumor parameters that may be obtained by an infrared (IR) camera. As a non-invasive method, the IR camera is a superior asset for the presented tumor detection method. Using a simple heat source model in training the ANN allows the system to measure a 0.01 °C temperature difference in an ideal condition and 0.03 °C temperature difference under a 10% noise condition.

The sensitivity of the parameter was studied along with the surface temperature sensitivity. According to the obtained results, the skin surface temperature is the most sensitive among all the parameters studied (i.e. depth, and intensity). Also, since the method measures a very low-temperature difference, it requires a highly sensitive and accurately calibrated IR camera system. Using such a camera system, an efficient way of finding tumor parameters was developed which relies only on the maximum temperature and the local temperature (the temperature at any location from the maximum temperature) on the skin surface. It is feasible to use this method since, in living systems we are dealing with maximum temperature.

According to the results, the methodology can help locate a tumor region on an external body part, which could be useful and important in studying tumor evolution after a treatment procedure.

The proposed methodology is able to detecte tumors that are located superficially. In case of abnormal and non symmetrical temperature detection, it is possible to combin the proposed method with other active tomography, to diagnose deep-seated tumors in case of temperature.

As this study was not validated with clinical data or biopsy results, the method could not be applied as a diagnostic tool. One of the future works includes animal and clinical tastings. In order to validate the proposed methodology, the ongoing research in Electro-thermal laboratory at Ryerson University is trying to build collaboration with Canadian Breast Cancer Society to collect the surgical data and true thermogram.

## Abbreviation

ANN: artificial neural network
